# CATH-2-derived antimicrobial peptide inhibits multidrug-resistant *Escherichia coli* infection in chickens

**DOI:** 10.3389/fcimb.2024.1390934

**Published:** 2024-05-15

**Authors:** Shihao Hao, Wenhui Shi, Liujun Chen, Tianyou Kong, Bin Wang, Shuming Chen, Xiaomin Guo

**Affiliations:** ^1^ College of Veterinary Medicine, Shanxi Agricultural University, Taigu, China; ^2^ Beijing Chest Hospital, Capital Medical University, Beijing, China

**Keywords:** Avian colibacillosis, MDR, *E. coli*, CATH-2, C2-2

## Abstract

Avian colibacillosis (AC), caused by infection with *Escherichia coli* (*E. coli*), is a major threat to poultry health, food safety and public health, and results in high mortality and significant economic losses. Currently, new drugs are urgently needed to replace antibiotics due to the continuous emergence and increasing resistance of multidrug-resistant (MDR) strains of *E. coli* caused by the irrational use of antibiotics in agriculture and animal husbandry. In recent years, antimicrobial peptides (AMPs), which uniquely evolved to protect the host, have emerged as a leading alternative to antibiotics in clinical settings. CATH-2, a member of the antimicrobial cathelicidin peptide family, has been reported to have antibacterial activity. To enhance the antimicrobial potency and reduce the adverse effects on animals, we designed five novel AMPs, named C2-1, C2-2, C2-3, C2-4 and C2-5, based on chicken CATH-2, the secondary structures of these AMPs were consistently α-helical and had an altered net charge and hydrophobicity compared to those of the CATH-2 (1-15) sequences. Subsequently, the antimicrobial activities of CATH-2 (1-15) and five designed peptides against MDR *E. coli* were evaluated *in vitro*. Specifically, C2-2 showed excellent antimicrobial activity against either the ATCC standard strain or veterinary clinical isolates of MDR *E. coli*, with concentrations ranging from 2-8 *μ*g/mL. Furthermore, C2-2 maintained its strong antibacterial efficacy under high temperature and saline conditions, demonstrating significant stability. Similarly, C2-2 retained a high level of safety with no significant hemolytic activity on chicken mature red blood cells or cytotoxicity on chicken kidney cells over the concentration range of 0-64 *μ*g/mL. Moreover, the administration of C2-2 improved the survival rate and reduced the bacterial load in the heart, liver and spleen during MDR *E. coli* infection in chickens. Additionally, pathological damage to the heart, liver and intestine was prevented when MDR *E. coli* infected chickens were treated with C2-2. Together, our study showed that C2-2 may be a promising novel therapeutic agent for the treatment of MDR *E. coli* infections and AC.

## 1 Introduction

Avian colibacillosis (AC) is an infectious disease caused by the avian pathogen *E. coli* (APEC), that results in fibrinoid lesions around internal organs (Gao et al., 2018; Nicolas et al., 2023). This disease can lead to primary infection as well as secondary infection with other viruses or pathogens (Sargeant et al., 2019; Walker et al., 2020). Antibiotics play an important role in the prevention and treatment of AC (Roth et al., 2019), but their overuse has led to the development of multidrug-resistant (MDR) *E. coli* strains (Kumar et al., 2020). By 2030, more than half of incoming *E. coli* isolates could be resistant to third-generation cephalosporins (Alvarez-Uria et al., 2018). This complicates the treatment of AC and presents a significant obstacle to the agricultural industry. Additionally, excessive antibiotic use is also linked to drug residues in soil, groundwater and animal-derived foods, posing a significant threat to human health (Ghimpeţeanu et al., 2022; Huang et al., 2022; Shengnan et al., 2023). Many countries have banned the use of antibiotics in animal feed to eliminate their adverse effects (Treiber and Beranek-Knauer, 2021; Getachew et al., 2023). Moreover, second, third and fourth generation antibiotics have been developed, most of which are chemically modified from the original antibiotics (Voulgaris et al., 2019; Rusu and Buta, 2021). Unfortunately, these new antibiotics do not fully address the problems associated with *E. coli* resistance because the antibacterial mechanisms remain unchanged. Therefore, it is necessary to develop new effective drugs against *E. coli* infections, especially for MDR strains.

Antimicrobial peptides (AMPs), which are components of the innate immune system, play a crucial role in defense against external pathogens (Torres et al., 2019; Ciulla and Gelain, 2023). AMPs are commonly present in various organisms and typically range from 12 to 50 amino acids, half of which contain arginine and lysine residues (Valdez-Miramontes et al., 2021). These peptides were primarily found on animal surfaces in close proximity to pathogens, including the skin, ears, eyes, and epithelial lining of the tongue, trachea, lungs, intestines, bone marrow, and testes (Hancock and Scott, 2000). AMPs have been reported to effectively suppress various microorganisms, such as gram-positive or gram-negative bacteria, fungi, parasites, viruses and tumor cells. The majority of AMPs directly target the bacterial cell membrane, producing antimicrobial effects (Mourtada et al., 2019), and some have multiple targets within the bacterial cell (Torres et al., 2019), a unique mechanism for killing MDR bacteria (Xuan et al., 2023). Subsequent studies have shown that AMPs have various additional functions, including inducing differentiation, and chemotaxis; activating various leukocyte types; inhibiting lipopolysaccharide-induced effects; promoting DNA uptake and phagocytosis; and promoting wound healing (Semple and Dorin, 2012; Mwangi et al., 2019). Hence, the development of AMPs as superior alternatives to antibiotics for the treatment of bacterial infections, especially those caused by drug-resistant bacteria, has great potential for expansion (Rima et al., 2021; Dong et al., 2022).

Chickens have three primary families of antimicrobial peptides: defensins, cathelicidins and LEAP-2. CATH-1 to CATH-3 and CATH-B1, members of the cathelicidin family, have been identified as four specific antimicrobial peptides (Goitsuka et al., 2007; Achanta et al., 2012). Among them, CATH-2, which is found mainly in heterophilic cells and consists of two helices (Cuperus et al., 2013), has been reported to have antibacterial and immunoregulatory properties (Coorens et al., 2017; van Harten et al., 2018; Scheenstra et al., 2019). Importantly, its antimicrobial activity is unaffected by salt concentration (Zhang and Sunkara, 2014), making it a valuable template for novel AMP design.However, the therapeutic use of CATH-2 is limited by its high cytotoxicity and relatively weak antibacterial activity (Askari et al., 2021; Botelho Sampaio de Oliveira et al., 2023). In this study, we further optimized CATH-2 (1-15), a peptide derived from CATH-2, by intercepting the core antibacterial region and reducing the cytotoxicity of the original sequence (van Dijk et al., 2009; Xiao et al., 2009). The antibacterial activity against *E. coli* and the stability and safety of five designed peptides, C2-1, C2-2, C2-3, C2-4 and C2-5, were subsequently evaluated *in vitro*. Surprisingly, compared with CATH-2 (1-15) and most other designed peptides, C2-2 showed superior antibacterial activity against standard strains and veterinary clinical MDR strains of *E. coli*. In addition, C2-2 maintained stable antibacterial activity at high temperatures and in saline solutions while maintaining a high level of safety within a specific concentration range. Furthermore, C2-2 administration significantly reduced the mortality, bacterial load and pathological damage induced by MDR *E. coli* infection in chickens. In conclusion, this study showed that C2-2 may be a potent candidate against standard and MDR *E. coli* infections.

## 2 Materials and methods

### 2.1 Animal

The 1-day-old chickens used in this study were obtained from Shanxi Jiabo Agricultural and Animal Husbandry Development Co., Ltd. The chicks were group-housed and given ad libitum access to food and water until 21 days of age.

### 2.2 Peptide design and synthesis

Five new AMPs were generated through the incorporation of amino acid substitutions into the CATH-2 (1-15) sequence. CATH-2 (1-15) (RFGRFLRKIRRFRPK), C2-1 (RFGRFLRKIRRFIPK), C2-2 (RFGRFLRKIRRMRLK), C2-3 (RFGRHLRKIIRFRIK), C2-4 (RFGRNLRKIRRFWPK), and C2-5 (RFGRFLRKIRFFRLK) were synthesized by PeptideValley. The purity of the products was determined to be greater than 95% by high-performance liquid chromatography (HPLC) and mass spectrometry.

### 2.3 Bioinformatics analysis

The physicochemical properties of the AMPs were analyzed using ProtParam software (https://web.expasy.org/protparam/). The spatial structure of the AMPs was constructed using PRBS (https://mobyle2.rpbs.univ-paris-diderot.fr/cgi-bin/portal.py#welcome), while helical wheel models of the resulting peptides were created using HeliQuest (https://heliquest.ipmc.cnrs.fr/).

### 2.4 Bacterial strains

*E. coli* (ATCC 8739) and clinical isolates of *E. coli* (*E1, E9, E12, E14, E16*, and *E18*) were stored at the Animal Biochemical Laboratory of Shanxi Agricultural University and grown in Luria-Bertani (LB) broth as previously reported (Zhao et al., 2021). Circular 6 mm paper pieces were prepared with filter paper and autoclaved for sterilization. Solutions of penicillin (PG), florfenicol (FF), ciprofloxacin (CIP), enrofloxacin (ENR), and gentamicin (GM) were prepared according to the Clinical and Laboratory Standards Institute (CLSI). Then, 0.5 mL of antibiotic solution was added to 100 sterilized papers. Subsequently, the paper pieces were fully infiltrated and dried in a vacuum drying oven to produce antibiotic susceptibility papers. The *E. coli* strain was retrieved from the -20°C freezer and activated by incubating it on LB agar media at 37°C for 24h. Individual colonies were selected and transferred to LB liquid media. Thereafter, the *E. coli* suspension was obtained by shaking at 37°C and 160 r/min for 18h. The obtained suspension was centrifuged at 5000 r/min for 5min, after which the supernatant was discarded, and the bacteria were resuspended in fresh medium to a concentration of 1×10^6^ CFU/mL. Two hundred microliters of the bacterial suspension mentioned above was spread evenly onto LB agar medium. Then, the antibiotic susceptibility papers were placed on the medium surface using sterile tweezers. After 24h of incubation at 37°C, the diameter of the inhibition zone was measured to determine the susceptibility of *E. coli* to antibiotics.

### 2.5 Antimicrobial activity assay *in vitro*

The minimum inhibitory concentrations (MICs) of the AMPs were determined using the microbroth dilution technique (van Harten et al., 2021). One hundred microliters of bacterial suspension at a concentration of 1×10^6^ CFU/mL was pipetted into a 96-well plate. Then, 100 *μ*L of AMP or gentamicin at various concentrations was added, while the bacterial suspension without AMPs and blank medium were used as controls. The plate was incubated at a constant temperature of 37°C for 18h, after which the turbidity of the wells was observed to determine the MIC. Fifty microlitres of liquid was extracted and plated on LB agar from wells exhibiting complete bacterial growth inhibition. The minimum bactericidal concentration (MBC) of the AMPs was assessed by counting colonies following a 24h incubation at 37°C.

### 2.6 Sterilization kinetics assay

The sterilization kinetics assay was performed following previous methods (Peng et al., 2022; Yong Fang et al., 2023). Gentamicin, C2-1 and C2-2 at a concentration of 8 *μ*g/mL each were added to a bacterial suspension of 1×10^5^ CFU/mL, and incubated at 37°C for 0, 15, 30, 60, 120, or 240min. At each time point, 10 *μ*L of sample was diluted in 1 mL of PBS, and 100 *μ*L of the dilution was plated on LB agar plates. After incubating at 37°C for 24h, the number of colonies was counted.

### 2.7 Stability assay

The MICs of the AMPs against *E. coli* (*ATCC 8739*) at different temperatures and physiological salt concentrations were evaluated. The thermal stability of the AMPs was assessed by incubating them at a concentration of 500 *μ*g/mL for 1h at 0, 37, and 100°C, using untreated AMPs as a control. The salt ion stability of the AMPs was determined by incubating them at a concentration of 500 *μ*g/mL in solutions of NaCl (150 mM), KCl (4.5 mM), MgCl2 (1 mM), and CaCl2 (2.5 mM) for 1h, using untreated samples as controls. The changes in the MICs of the AMPs were assessed following these experimental procedures.

### 2.8 Hemolytic activity assay

Fresh blood was harvested from chickens, anticoagulated with 0.2% sodium heparin, and centrifuged at 1000 r/min for 10min to remove the supernatant. The lower erythrocytes were collected, gently washed twice with sterile PBS, and subsequently prepared as a 2% erythrocyte suspension. The erythrocyte suspension was mixed with AMPs at different concentrations in equal volumes. Erythrocytes treated with PBS served as a negative control, while erythrocytes treated with 2% Triton X-100 served as a positive control. The mixture was incubated at 37°C for 1h, after which the absorbance at 570 nm was measured using a multifunctional enzyme marker.


$$\text{Hemolysis ratio }\left(\%\right)=\frac{\text{OD peptide treatment}-\text{OD negative control}}{\text{OD positive control}-\text{OD negative control}}\times 100\%$$

### 2.9 Cytotoxicity assay

Chicken renal cells were trypsinized, seeded at 5000 cells per well in 96-well plates, and incubated for 24h. The cell culture medium was subsequently discarded, and AMPs were added at different concentrations to the wells, which were subsequently filtered through a 0.22 *μ*m filter to eliminate bacteria. The mixture was incubated for another 24h, after which 50 *μ*L of 3-(4,5-dimethyl-2-thiazolyl)-2, 5-diphenyl tetrazolium bromide (MTT) reagent (5 mg/mL) was directly added to each well. The mixture was incubated for 4h. Then 50 *μ*L of MTT reagent (5 mg/mL) was added directly to each well and incubated for another 4h. To dissolve the crystals, 150 *μ*L of dimethyl sulfoxide (DMSO) was added, and the mixture was vortexed for 10min. The absorbance was measured at 490 nm using a multifunctional enzyme marker.

### 2.10 Chicken infection with MDR *E. coli*

Fifty 21-day-old chickens were randomly divided into five groups: blank control (500 *μ*L of normal saline), *E. coli* infection (500 *μ*L of normal saline), C2-2 treatment (200 *μ*g/mL, 500 *μ*L), gentamicin treatment (200 *μ*g/mL, 500 *μ*L) and enrofloxacin treatment (200 *μ*g/mL, 500 *μ*L). Except for the blank control group injected with 500 *μ*L of normal saline, the other four groups were injected with 500 *μ*L of 3×10^9^ CFU/mL *E. coli* (*E16*) into the pectoral muscles. Then, normal saline, C2-2, gentamicin and enrofloxacin were injected through the pectoral muscles 4h after *E. coli* infection according to the above methods. Clinical symptoms and were observed and documented for the chickens in each group after infection. After 72h, the chickens in each group were euthanized and examined for internal organ damage. Paraffin sections were prepared for microscopic examination of pathological changes. Under sterile conditions, 1 g samples of heart, liver, and spleen were collected, and each sample was supplemented with 20 mL of sterile PBS. After thorough grinding, the sample was diluted to the appropriate multiple, and 100 *μ*L of the diluent was spread onto LB agar medium. The plates were incubated for 24h, after which the colonies were counted.

### 2.11 Statistical analysis

Statistical analysis was performed using one-way analysis of variance (ANOVA). The data represent three independent experiments and are presented as mean ± SEM. A p-value of< 0.05 was considered to indicate statistical significance.

## 3 Results

### 3.1 Physicochemical characterization of the designed peptides

**Figure 1:**
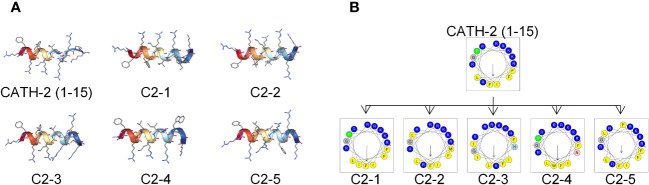
Structure prediction of AMPs. **(A)** Prediction of the spatial structure of AMPs. **(B)** Helical wheel projections of AMPs. Residues are color-coded: polar basic residues in blue, non-polar hydrophobic residues in yellow, glycine in grey, and proline represented by green circles. The hydrophobic moment is marked by a black arrow on the helical wheel.

The sequences and physicochemical properties of the peptides used are listed in **Table 1**. CATH-2 (1-15) is an N-terminal truncated peptide of chicken CATH-2 that consists of 15 amino acids and has an α-helical secondary structure and 8 positive charges. Based on the CATH-2 (1-15) sequence, five peptides, named C2-1 to C2-5, were designed by substituting amino acids to modify their charge and hydrophobicity. C2-2 was positively charged with 8 net charges, and the others had 7 net charges. Among them, CATH-2 (1-15) had the lowest hydrophobicity (0.103), and C2-5 had the highest hydrophobicity (0.355). According to the average hydrophilicity, C2-4 had the strongest hydrophilicity, followed by CATH-2 (1-15), and C2-2 ranked third. The predicted secondary structures showed that all five peptides were α-helical (**Figure 1A**).The spiral-wheel model highlighted changes in the hydrophobic moment, as well as the hydrophobic and hydrophilic characteristics of the AMPs (**Figure 1B**). All six peptides had an amphipathic structure that formed a hydrophilic and hydrophobic side.

**Table 1:** Physicochemical properties of the designed peptides.

Peptides	Sequence	NC	MW (Da)	GRAVY	H	HM (*µ*M)
CATH-2 (1-15)	RFGRFLRKIRRFRPK	+8	2033.51	-1.340	0.103	0.585
C2-1	RFGRFLRKIRRFIPK	+7	1990.48	-0.740	0.291	0.754
C2-2	RFGRFLRKIRRMRLK	+8	2033.56	-1.040	0.131	0.539
C2-3	RFGRHLRKIIRFRIK	+7	1966.48	-0.733	0.252	0.577
C2-4	RFGRNLRKIRRFWPK	+7	2030.46	-1.520	0.161	0.713
C2-5	RFGRFLRKIRFFRLK	+7	2040.54	-0.493	0.355	0.360

NC, Net charge; MW, molecular weight; GRAVY, Grand average of hydropathicity; H, Hydrophobicity; HM, Hydrophobic moment.

### 3.2 Antibacterial activity of the designed peptides *in vitro*

To comprehensively and accurately evaluate the effects of AMPs on different strains of *E. coli*, we isolated 6 strains of *E. coli* (*E1, E9, E12, E14, E16*, and *E18*) from poultry farms in Shanxi Province and analyzed their resistance to penicillin, florfenicol, ciprofloxacin, enrofloxacin and gentamicin, which are commonly used in the aquaculture industry for the treatment of *E. coli* infections. The results indicated that the 6 *E. coli* strains were all resistant to penicillin, florfenicol and enrofloxacin. Moreover, only the *E14* strain of *E. coli* was sensitive to ciprofloxacin, while three strains (*E12, E14*, and *E16*) of *E. coli* were susceptible to gentamicin. In summary, E. coli strains E1 and E18 were found to have relatively high levels of resistance (**Table 2**).

**Table 2:** Drug-sensitivity test.

*E. coli*	penicillin	florfenicol	ciprofloxacin	enrofloxacin	gentamicin
ATCC 8739	S	S	S	S	I
E1	R	R	R	R	R
E9	R	R	R	R	I
E12	R	R	R	R	S
E14	R	R	S	R	S
E16	R	R	R	R	S
E18	R	R	R	R	R

R, resistant strains; I, intermediate strains; S, sensitive strains.

The antimicrobial activities of CATH-2 (1-15) and the designed peptides were tested against 7 strains of *E. coli*. C2-1 and C2-2 exhibited potent antibacterial activity against 6 MDR *E. coli* strains. The MICs of C2-1 ranged from 0.5 to 16 *μ*g/mL, and 0.5 *μ*g/mL could inhibit the growth of *E9*. The MICs of C2-2 ranged from 2 to 8 *μ*g/mL. Compared with the CATH-2 (1-15), it increased the inhibitory effect on six MDR *E. coli* strains by 2 to 32 times. The inhibitory effects of C2-3 and C2-5 were slightly weaker than those of C2-1 and C2-2. The inhibitory effect of C2-4 was lower than that of the CATH-2 (1-15), which had the poorest inhibitory effect. The MICs of gentamicin were between 4- and 64 *μ*g/mL (**Table 3**). The MBC values of the AMPs further confirmed the above results (**Table 4**). In brief, the MBC values of CATH-2 (1-15) and the designed peptides were found to be greater than the MIC values. It is worth noting that the MBC values of C2-2 remained within the range of 2-8 *μ*g/mL. The results suggested that C2-1 and C2-2 exhibited potent *in vitro* inhibitory effects on several MDR *E. coli* strains.

**Table 3:** The MIC (*μ*g/mL) values of AMPs against *E. coli* strains.

*E. coli*	CATH-2 (1-15)	C2-1	C2-2	C2-3	C2-4	C2-5	gentamicin
ATCC 8739	64	2	8	16	16	1	8
E1	128	16	4	32	128	8	64
E9	4	0.5	2	2	64	64	8
E12	8	1	2	1	16	8	4
E14	64	16	8	8	32	8	4
E16	32	16	8	16	64	16	4
E18	64	2	4	16	128	16	64

**Table 4:** The MBC (*μ*g/mL) values of AMPs against *E. coli* strains.

*E. coli*	CATH-2 (1-15)	C2-1	C2-2	C2-3	C2-4	C2-5	gentamicin
ATCC 8739	64	4	8	16	16	1	8
E1	>128	16	8	64	128	8	64
E9	4	1	2	4	128	128	8
E12	8	1	2	1	32	8	4
E14	128	16	8	8	64	8	4
E16	64	64	8	64	64	16	4
E18	64	4	4	32	128	32	128

**Figure 2:**
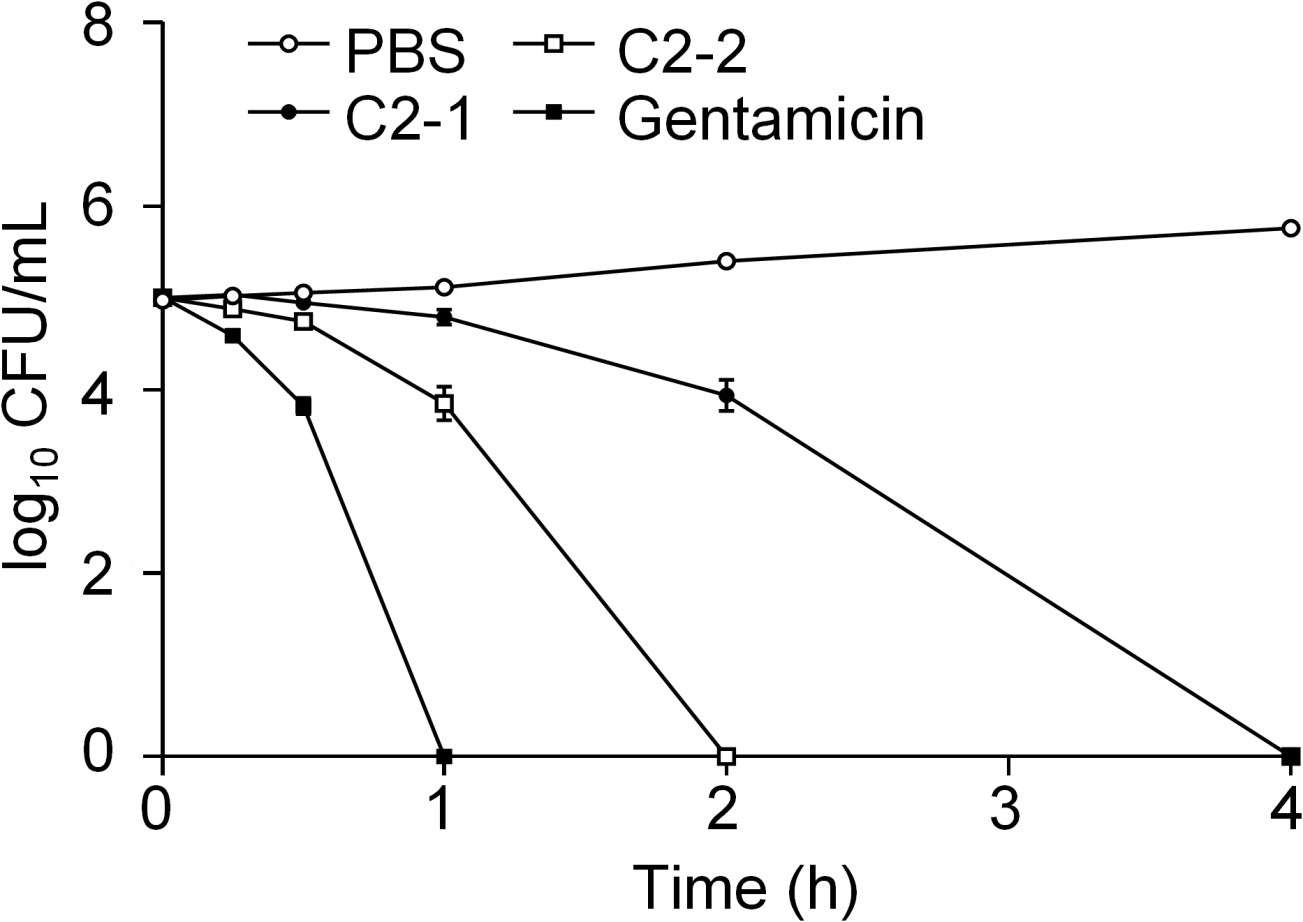
Bacterial survival after treatment with peptides. The bacterial suspension of *E. coli* (ATCC 8739) was incubated with C2-1 and C2-2, each at a concentration of 8 *μ*g/mL of gentamicin, for 4h at 37°C to determine the number of viable bacteria. Data represent three independent experiments and are presented as mean ± SEM.

In this study, the bactericidal kinetics of C2-1, C2-2, and gentamicin were evaluated against *E. coli* (*ATCC8739*), indicating their efficacy against bacteria. Gentamicin at a concentration of 8 *μ*g/mL exhibited the most potent bactericidal effect, as it completely eradicated bacteria within 60min. C2-2 showed the second most potent bactericidal activity, while C2-1 had the least potent effect, requiring 240min for complete bacterial eradication (**Figure 2**).

### 3.3 Stability and safety of the designed peptides

The thermal stability and salt ion stability of AMP are two important factors limiting its clinical use and were also tested in this study. As shown in **Table 5**, after 1h of incubation at 100°C, CATH-2 (1-15) exhibited a 2-fold increase in antimicrobial efficacy, whereas C2-5 experienced a 4-fold reduction under the same conditions, while the MICs of the other four designed peptides remained constant. In addition, antimicrobial activity of CATH-2 (1-15) increased 2-fold in the presence of sodium ions and fourfold in the presence of potassium and magnesium ions. C2-1 demonstrated a 4-fold increase in antimicrobial activity with sodium ions but a fourfold decrease with calcium ions. C2-2 displayed a 16-fold increase in antimicrobial activity with sodium ions, potassium ions, or magnesium ions and a 2-fold increase with calcium ions. C2-3 experienced a 4-fold increase in antimicrobial activity with potassium or magnesium ions. C2-4 exhibited a 2-fold decrease in antimicrobial activity with sodium, potassium, or calcium ions, while C2-5 exhibited a fourfold decrease with sodium, potassium, or magnesium ions and an eightfold decrease with calcium ions. In general, with the exception of C2-4 and C2-5, the other analogs consistently retained potent antimicrobial activity at diverse temperatures or under salt ion conditions.

**Table 5:** The MIC (*μ*g/mL) values of AMPs against *E. coli* (ATCC 8739) at different temperatures and salt solutions.

AMPs	Thermal Stability			Salt Stability			
	0°C	37°C	100°C	NaCl (150 mM)	KCl (4.5 mM)	MgCl2 (1 mM)	CaCl2 (2.5 mM)
CATH-2 (1-15)	64	64	32	32	16	16	64
C2-1	2	2	2	0.5	2	2	8
C2-2	8	8	8	0.5	0.5	0.5	4
C2-3	16	16	8	16	4	4	8
C2-4	16	16	16	32	32	16	32
C2-5	1	1	4	4	4	4	8

**Figure 3:**
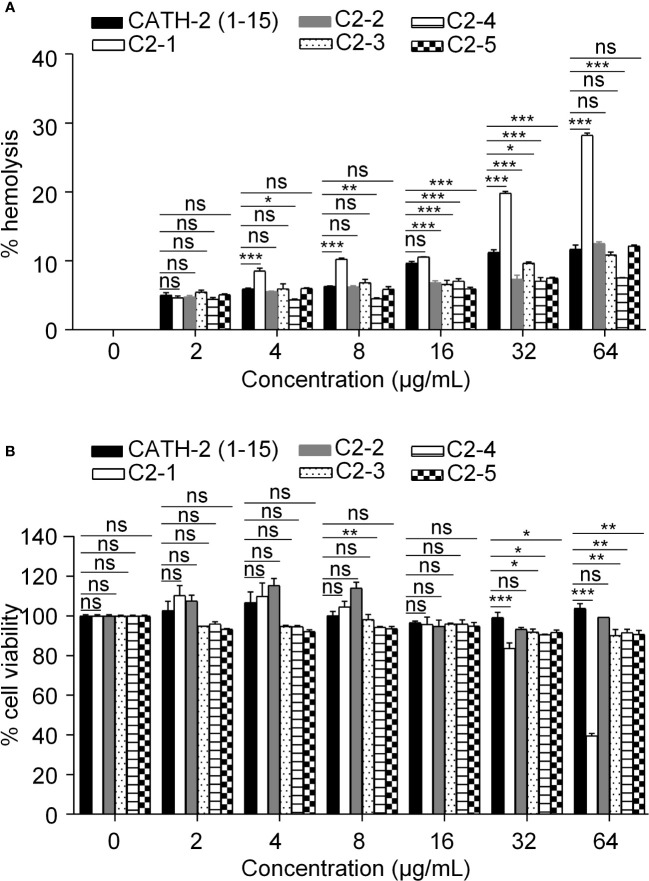
Safety of the designed peptides. **(A)** Hemolytic activities of the peptides at various concentrations upon incubation with chicken erythrocytes for 24h. **(B)** Cytotoxicity of the peptides at various concentrations upon incubation with chicken kidney cells for 24h. Data represent three independent experiments and are presented as mean ± SEM. *P < 0.05; **P < 0.01; ***P < 0.001; ns, not significant.

In this study, we investigated the hemolytic activity of peptides derived from CATH-2 on mature chicken erythrocytes. Except for C2-1, the remaining four peptides exhibited no significant hemolytic activity compared to that of CATH-2 (1-15) (**Figure 3A**). In addition, cytotoxicity is an important indicator of the safety of AMPs. Except for C2-1, no significant cytotoxicity against chicken kidney cells was observed after treatment with the designed peptides in the concentration range of 0-64 *μ*g/mL (**Figure 3B**).

### 3.4 C2-2 inhibits MDR-related *E. coli* infection in chickens

**Figure 4:**
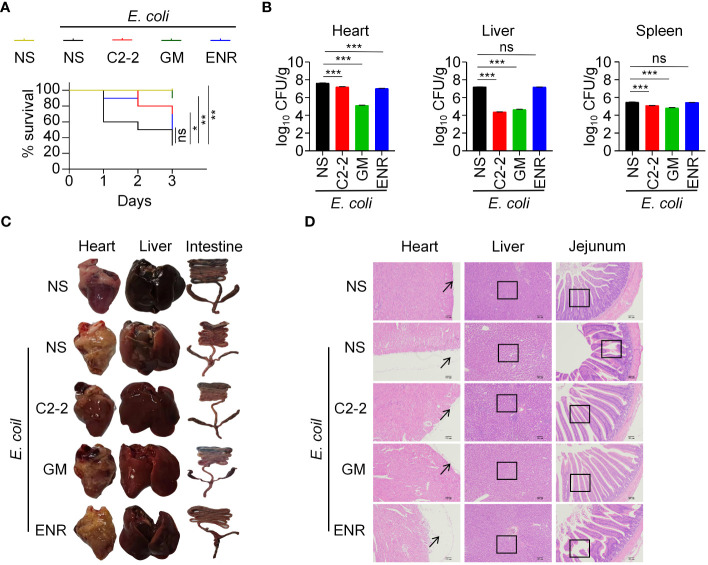
C2-2 inhibits MDR *E. coli* infection *in vivo*. **(A)** Protective efficacy in chickens in each group after infection. **(B)** Organ bacterial load in the *E. coli* infection model. **(C)** Pathological changes of heart, liver and intestine in different treatment groups. **(D)** Pathological changes in heart, liver and jejunum of chickens in each experimental group. NS: normal saline; GM: gentamicin; ENR: enrofloxacin. Data represent three independent experiments and are presented as mean ± SEM. *P < 0.05; **P < 0.01; ***P < 0.001; ns, not significant.

The *E16* strain of *E. coli* was selected for chicken infection based on its resistance and because a previous study reported that *E16* exhibited greater toxicity. To determine the role of C2-2 in preventing *E. coli* infection *in vivo*, chickens were infected with 1.5× 10^9^ CFU of *E16* with or without C2-2 administration, and survival was monitored over time. The blank control group showed no abnormal changes after 3 days of infection and maintained 100% survival. In contrast, the *E. coli* infection group exhibited the most severe clinical symptoms, such as depression, loss of appetite, drooping wings, sensitivity to cold, and discharge of yellowish-green feces, which were present 12h post infection. Mortality of normal infected chickens occurred at 1 day post infection, whereas infected chickens treated with C2-2 began to die at 2 days post infection. In addition, the survival rate of *E. coli* infected chickens in the presence of C2-2 was 70% by day 3, which was markedly greater than that of chickens not receiving C2-2 administration (30%) (**Figure 4A**). The 3-day mortality rate of the C2-2 treatment group was intermediate between that of the gentamicin and enrofloxacin treatment groups at 10% and 50%, respectively. There was no difference in survival rate between the enrofloxacin-treated and untreated groups, and the survival rate in both groups was significantly lower than that in the gentamicin-treated group, which was due to the presence of gentamicin-sensitive and enrofloxacin-resistant *E16* strains (**Figure 4A**). To investigate whether the attenuation of pathogenesis noted above was due to a decreased burden of *E. coli* in the chickens administered C2-2, we analyzed the bacterial loads in the heart, liver and spleen after 3 days of infection. The results showed that C2-2 and gentamicin significantly reduced the bacterial loads in the heart, liver and spleen of infected chickens, whereas enrofloxacin affected only the heart bacterial load. The liver bacterial burden in the *E. coli* infection group was 1.61×10^7^ CFU/g, and the liver bacterial load in the C2-2 treatment group was 2.37×10^4^ CFU/g, which was reduced by approximately 680 times, indicating that C2-2 treatment had the greatest effect on the bacterial load in the liver (**Figure 4B**).

After the chickens died, an autopsy was conducted to examine the pathological changes in the heart, liver, and intestine. Chickens infected with MDR *E. coli* had a heart surface covered with a yellow fibrinous exudate. The liver was enlarged, tender in texture, yellow in color, and occasionally covered with yellow-white cellulose exudate. In the intestines of the chickens, congestion, hemorrhage and enlargement of the mesenteric lymph nodes were observed to varying degrees. Some of the affected chickens exhibited symptoms of splenomegaly and severe thymic congestion. Overall, in contrast to those in the *E. coli* infection group, there was a significant reduction in organ lesions in both the C2-2 and gentamicin treatment groups (**Figure 4C**). The histologic sections of the heart, liver and jejunum from the different treatment groups are presented in **Figure 4D**. Compared with tose in the negative control group, the *E. coli* infection group and the enrofloxacin treatment group exhibited significant thickening of the external cardiac membrane, extensive fibrous coverage on the surface, swelling, and rupture of myocardial fibers, as well as fibrous exudates in the interstitial spaces. Hepatocytes showed swelling and granular degeneration, accompanied by disordered arrangement of hepatocyte cords and marked infiltration of inflammatory cells. The jejunal villi were observed to be disrupted, and the intestinal mucosa was congested and swollen with infiltration of inflammatory cells. In the C2-2 treatment group and the gentamicin treatment group, the myocardial fibers were arranged neatly without any granular degeneration of cells, with only a small amount of fibrinous exudate on the surface. The liver lobules appeared normal in structure with visible hepatocytes and no granular degeneration. The jejunal villi were relatively structurally intact with a small number of inflammatory cells in the submucosa.

## 4 Discussion

AC caused by *E. coli* infection is associated with respiratory and systemic diseases (Alber et al., 2021). The primary treatment for AC involves antibiotics, which contribute to the increasing occurrence of MDR *E. coli* (Christensen et al., 2021). In this study, drug susceptibility testing was performed on clinical isolates of *E. coli*, revealing their MDR nature. This finding confirms the widespread presence of MDR *E. coli*, which poses a significant threat to the global agricultural industry and emphasizes the need to develop drugs to combat drug-resistant bacterial infections.

The antimicrobial activity of AMPs primarily depends on the physical and structural characteristics of the peptide, including its net charge, hydrophobicity, amphiphilicity, and sequence length (Torres et al., 2019). Minor alterations in the amino acid sequence can entirely alter the physical and chemical properties of an AMP. In particular, the net charge and hydrophobicity are thought to influence bacterial binding. Bacterial cell membranes, which are mainly lipid-based, carry a substantial negative charge and can interact with positively charged AMPs (Dwivedi et al., 2023). Nevertheless, excessive hydrophobicity can result in increased hemolytic activity. Peptide amphiphilicity closely correlates with the hydrophobic moment, which, in turn, is directly influenced by the secondary structure of the peptide (Eisenberg et al., 1982). Thus, the structure of the peptide is critical for antimicrobial activity. CATH-2 (1-15) has shown antimicrobial activity against a wide range of bacteria (Sharma et al., 2022). However, its antibacterial activity is relatively weak, and its effectiveness against MDR *E. coli* has not been thoroughly investigated. This study aimed to modify the amino acid composition of CATH-2 (1-15) to regulate hydrophobicity and charge, leading to the design of five AMPs. The feasibility of using these AMPs as antibiotic replacements for the treatment of MDR *E. coli* infections was investigated.

Among the six AMPs evaluated in this study, C2-4 had the lowest net charge and hydrophobicity, resulting in the weakest antimicrobial activity. The MICs of CATH-2 (1-15) and C2-2 against MDR *E. coli* were 4-128 *μ*g/mL and 2-8 *μ*g/mL, respectively. This difference may be attributed to the similar net charges of CATH-2 (1-15) and C2-2, but C2-2 possessed greater hydrophobicity than CATH-2 (1-15). Compared to C2-2, C2-1 had a slightly lower net charge but greater hydrophobicity and amphiphilicity, resulting in enhanced antimicrobial activity. However, the elevated hydrophobicity of C2-1 was also associated with a significant increase in hemolytic activity, which limited its clinical application. These findings suggest that the antimicrobial activity of peptides is not determined by a specific amino acid sequence or physicochemical property. Instead, it is related to the overall amino acid composition of the peptide (Findlay et al., 2010).

*In vitro* bacterial inhibition experiments revealed that the MICs of C2-2 against MDR *E. coli* ranged from 2 to 8 *μ*g/mL. The difference in the inhibitory concentration exhibited by the AMPs against different strains of bacteria may be attributed to the differences in the membrane lipid composition of different MDR strains, drug-resistant genes, the intracellular environment, and other factors, which may affect the combination of the AMPs with the bacteria to exert antimicrobial effects. Additionally, at a concentration of 8 *μ*g/mL, C2-2 entirely eradicated *E. coli* within 2h, demonstrating a faster sterilization rate. Given the complex that AMPs encounter upon entering the body, this study examined changes in the MICs of AMPs under adverse conditions such as low temperature, high temperature, and elevated salt ion concentrations. Normally, the positive charge carried by salt ions affects the binding of AMPs to bacteria, thereby influencing antimicrobial activity (Shenrui et al., 2023). Notably, C2-2 exhibited strong antimicrobial activity even in these challenging environments, consistent with previous findings (Xiao et al., 2006). This is because C2-2 is unaffected by salt ions, which can impair its antistructural properties, and metal ions are themselves bacteriostatic, disrupting cell membranes and facilitating the bacteriostatic effects of the AMPs.

Previous studies have shown that treatment of chicken embryos with D-CATH-2, an analog of CATH-2, results in a significant reduction in the number of pathogenic *E. coli* and in the respiratory bacterial load (Cuperus et al., 2016). Our present study produced similar results, as treatment of MDR *E. coli*-infected chicks with C2-2 significantly improved the survival rate of infected chicks and markedly reduced bacterial counts in the hearts, livers, and spleens of the affected chicks. This study developed a low-toxicity, efficient, and stable AMP, providing a foundation for the development of drugs targeting MDR *E. coli*. However, the inhibitory mechanism of C2-2 against MDR *E. coli* and its immunomodulatory effects on this organism remain unclear and warrant further investigation.

## Data availability statement

The original contributions presented in the study are included in the article/supplementary material, Further inquiries can be directed to the corresponding authors.

## Ethics statement

The animal study was approved by the Animal Care Committee of Shanxi Agricultural University. The study was conducted in accordance with the local legislation and institutional requirements.

## Author contributions

XG: Methodology, Writing – original draft. SH: Methodology, Writing – original draft. WS: Resources, Writing – review & editing. LC: Investigation, Writing – review & editing. TK: Investigation, Writing – review & editing. BW: Resources, Writing – review & editing. SC: Conceptualization, Funding acquisition, Writing – review & editing.
